# Supplementary material for: Successional Shifts in Soil C:N:P stoichiometry and its environmental controls during forest restoration in Huize County, Qujing, Yunnan Province
Source: PLoS One. 2026 Aug 21;21(8):e0356261. doi: 10.1371/journal.pone.0356261 (PMC13496410; doi:10.1371/journal.pone.0356261)
Supplement: Supplementary file 2 [file pone.0356261.s004.zip › Data and Code/RDA/Pinus yunnanensis forest/RDA-3.pdf]

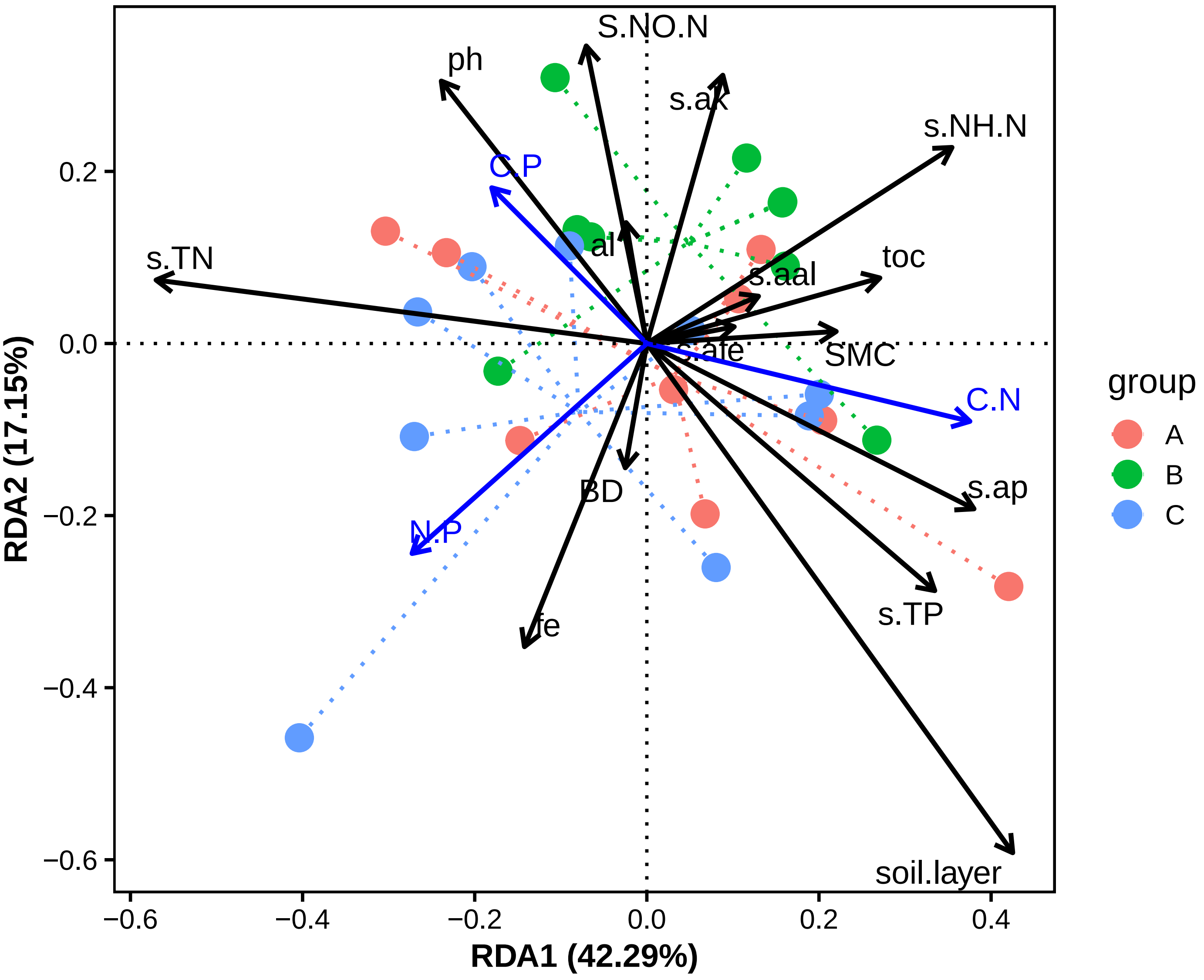
S.NO.N
ph
s.ak
s.NH.N
C.P
al
s.TN
toc
s.aal
s.afe
SMC
C.N
BD
N.P
s.ap
fe
s.TP
soil.layer
0.2
0.0
−0.2
−0.4
−0.6
−0.6
−0.4
−0.2
0.0
0.2
0.4
RDA2 (17.15%)
RDA1 (42.29%)
group
A
B
C
